# Age-dependent degeneration of an identified adult leg motor neuron in a *Drosophila* SOD1 model of ALS

**DOI:** 10.1242/bio.049692

**Published:** 2020-10-21

**Authors:** Anthony Agudelo, Victoria St. Amand, Lindsey Grissom, Danielle Lafond, Toni Achilli, Asli Sahin, Robert Reenan, Geoff Stilwell

**Affiliations:** 1 Department of Biology, Rhode Island College, 600 Mt. Pleasant Ave., Providence, RI, 02908 USA; 2 Department of Molecular Biology, Cell Biology & Biochemistry, Brown University, Providence, RI, 02912 USA

**Keywords:** ALS, Superoxide dismutase, *Drosophila* disease model, Neurodegeneration, Neuromuscular junction

## Abstract

Mutations in superoxide dismutase 1 (SOD1) cause familial amyotrophic lateral sclerosis (ALS) in humans. ALS is a neurodegenerative disease characterized by progressive motor neuron loss leading to paralysis and inevitable death in affected individuals. Using a gene replacement strategy to introduce disease mutations into the orthologous *Drosophila sod1* (*dsod1*) gene, here, we characterize changes at the neuromuscular junction using longer-lived *dsod1* mutant adults. Homozygous *dsod1^H71Y/H71Y^* or *dsod1^null/null^* flies display progressive walking defects with paralysis of the third metathoracic leg. In dissected legs, we assessed age-dependent changes in a single identified motor neuron (MN-I2) innervating the tibia levitator muscle. At adult eclosion, MN-I2 of *dsod1^H71Y/H71Y^* or *sod1^null/null^* flies is patterned similar to wild-type flies indicating no readily apparent developmental defects. Over the course of 10 days post-eclosion, MN-I2 shows an overall reduction in arborization with bouton swelling and loss of the post-synaptic marker *discs-large* (*dlg*) in mutant *dsod1* adults. In addition, increases in polyubiquitinated proteins correlate with the timing and extent of MN-I2 changes. Because similar phenotypes are observed between flies homozygous for either *dsod1^H71Y^* or *dsod1^null^* alleles, we conclude these NMJ changes are mainly associated with *sod* loss-of-function. Together these studies characterize age-related morphological and molecular changes associated with axonal retraction in a *Drosophila* model of ALS that recapitulate an important aspect of the human disease.

This article has an associated First Person interview with the first author of the paper.

## Abstract (precis)

**Summary:** A *Drosophila* gene-replacement model of ALS exhibits age-dependent dismantling of the neuromuscular junction.

## INTRODUCTION

Amyotrophic lateral sclerosis (ALS) is an adult onset neurodegenerative disease characterized by the progressive loss of motor neurons (Boillee et al., 2006). Post-diagnosis survival times average 3–5 years and currently there is no treatment available that substantially extends life despite the availability of two therapeutic drugs, riluzole and edaravone (Sawada, 2017). Much of our understanding of disease mechanisms derives from the study of dominant, single gene mutations causing familial ALS (fALS), which represents 10% of the total disease population. To date, 16 genes have been identified with clear causality to fALS (Taylor et al., 2016). Mechanistically, these gene products are involved in a range of cellular processes including autophagy, RNA metabolism, cytoskeletal transport, oxidative stress response and inflammation (reviewed in Taylor et al., 2016 and Nguyen et al., 2018). Together these genes help to define pathways important for disease pathogenesis.

Over 150 mutations in superoxide dismutase (SOD1) cause fALS (http://alsod.iop.kcl.ac.uk/) (Forsberg et al., 2019; Wroe et al., 2008). To date, all mutations increase the potential of the normally dimeric SOD1 to form destabilized monomers and aggregates (Lang et al., 2015). SOD1 fibrillization kinetics show strong, inverse correlations with disease duration (Lang et al., 2015; McAlary et al., 2016). Transgenic mouse models expressing mutant human SOD1 (hSOD1), accumulate hSOD1 positive aggregates in the cytoplasm and mitochondria of motor neurons (MNs). These animals also show age-dependent locomotor impairment, progressive paralysis and MN degeneration. Specifically, fast-fatigable MNs are lost first by apoptotic or necroptotic cell death mechanisms involving bcl-2/bax or ripk1 pathways (Morrice et al., 2017).

Although SOD1 is expressed ubiquitously and abundantly, restricted expression of mutant hSOD1 in either muscle or neurons can reproduce MN loss in mouse models (Dobrowolny et al., 2008; Jaarsma et al., 2008; Wong and Martin, 2010). These data suggest that neuromuscular junction (NMJ) maintenance, which requires signaling between the neuron, glia and muscle at the tripartite synapse is important for disease initiation and progression. The ‘dying-back’ of axons is an early feature of disease and is observed at pre-symptomatic stages (Fischer et al., 2004). Also, denervation precedes accumulation of SOD1-positive aggregates and is associated with mitochondrial vacuolization (Gould et al., 2006). Taken together, these results indicate that changes at the NMJ are early and critical features of ALS pathogenesis.

Although the various SOD1 models of ALS reproduce many disease features, they rely on overexpression of mutant hSOD1 in a genetic background, which includes endogenous expression of wild-type sod in the model organism. In mice, multiple copies of mutant hSOD1 are required to generate disease-related phenotypes and overexpression of wild-type hSOD1 alleles produced disease-like phenotypes including altered mitochondrial morphology, motor neuron degeneration, ataxia and shortened life span (Graffmo et al., 2013; Jaarsma, 2006; Jaarsma et al., 2000). A fly overexpression model produced similar results in which wild-type hSOD1 or the mutant variants G85R or A4V were driven in MNs using the bipartite Gal4/UAS system (Watson et al., 2008). Although expression of mutant hSOD1 generally produced more severe phenotypes, aged *D42-Gal4>UAS-hSOD1^+^* flies showed progressive locomotor defects and electrophysiological defects in the giant fiber pathway innervating the DLM and TTMs. These results raise the important question of the extent to which ALS model phenotypes are caused by an artifact of overexpression versus presence of disease-causing mutations.

Previously, we characterized a novel *Drosophila* ALS model created by a gene-replacement strategy (Sahin et al., 2017). Using ends-out homologous recombination, we replaced *dsod1^+^* with ALS-associated mutations including G85R, H48R or H71Y. Each of these three alleles produced recessive phenotypes that ranged in severity from early adult lethality prior to eclosion for *dsod1^G85R/G85R^* to shortened lifespan in *dsod1^H71Y/H71Y^* mutants (Sahin et al., 2017). Interestingly, recessive ALS-like phenotypes are seen in canine and murine models in which endogenous *sod* is mutated, and also in a G85R-YFP transgenic mouse model (Wang et al., 2009; Joyce et al., 2015; Katz et al., 2017). As adults, *dsod1^H71Y/H71Y^* homozygotes show shortened life span and progressive locomotor defects; however, there is no readily apparent MN loss based on TUNEL labeling studies or cell counts using nuclear-localized GFP expressed in glutaminergic neurons. Similar preservation of MNs is generally observed in G85R homozygotes; however, these flies die as pharate adults preventing the study of progressive changes in the adult central nervous system (CNS) (Sahin et al., 2017).

Although no MN loss was detected previously, we sought to determine whether there are distal changes in MN morphology over time in *dsod1*-mutant flies. Although the patterning, structure and function of the *Drosophila* NMJ is well established for identified MNs in larvae, the larval NMJ is maintained for only a short time before neuronal and muscle remodeling occurs after puparium formation (Brown et al., 2006; Watts et al., 2003). Therefore, the study of slow degenerative changes superimposed upon rapid development of larvae into pupae presents a challenge in well-characterized larval MNs. Alternatively, the study of adult neurons has focused mainly on non-motor neuron cell types including photoreceptor cells, CNS interneurons, olfactory or gustatory neurons or neurosecretory cell populations (for example, see Hussain et al., 2018; Logan et al., 2012). Recent work has begun characterizing adult MNs that innervate the abdomen, the proboscis or the leg (Hebbar and Fernandes, 2004; Hebbar et al., 2006; Mahoney et al., 2014). Degeneration of leg motor and sensory neuron populations have been studied in the context of neurodegeneration (Sreedharan et al., 2015). This work identified *Shaggy/GSK3*, *Hat-trick* and *Xmas-2* as suppressors of TDP-43 induced toxicity using genetic mosaics to drive mutant TDP-43 in restricted cell populations. In addition, Fernius et al. (2017) used leg motor and sensory neurons to profile degenerative phenotypes for various neurodegenerative diseases created by overexpressing various mutant alleles (Fernius et al., 2017). These studies relied on imaging MNs through the cuticle using GFP expressing lines coupled with relevant gene mutations.

Here we characterize changes in an identified adult MN (MN- I2) in dissected adult legs using standard immunohistochemical techniques and thus eliminating the need for complex genetic backgrounds with fluorescent tags to label structures. The adult leg of *Drosophila* is comprised of approximately 50 MNs that innervate the musculature to drive coordinated walking behavior (Baek and Mann, 2009; Brierley et al., 2009, 2012; Enriquez et al., 2015). The leg MNs arise from 11 neuroblast lineages and produce stereotyped axonal and dendritic arborization patterns (Baek and Mann, 2009). Our results show that MN- I2 axon morphology is normal in newly eclosed *dsod1^H71Y/H71Y^* homozygotes but undergoes progressive changes resulting in highly disorganized axonal projections with an overall reduction in axonal branch length and synaptic bouton number. In aged *dsod1^H71Y/H71Y^* animals, we find ‘ghost’ branches and associated boutons that lack the postsynaptic marker DLG. These changes reproduce several key features of the dismantling of the NMJ seen in ALS. Furthermore, similar phenotypes seen in *dsod1^null/null^* aged-adults suggest these changes result from SOD1 loss-of-function. Finally, this work establishes a model to study slow progressive changes in an identified MN over time.

## RESULTS

Denervation is a prominent feature of ALS, characterized by reduction in motor units and synapses leading to muscle wasting and eventually paralysis. Changes at the NMJ can be observed at presymptomatic stages in animal models and therefore represent early events in disease pathogenesis (Hegedus et al., 2007, 2008; Martineau et al., 2018). In *Drosophila* genetic ALS models, presynaptic changes have been characterized for mutant human *fused in sarcolemma* (FUS) driven within neurons. These flies showed altered active zone organization and concomitant decreases in evoked post-synaptic currents (Shahidullah et al., 2013). In *TAR DNA-binding protein-43 homolog* (*tbph*) mutants, decreased locomotion, loss of presynaptic terminals, and loss of MN populations were observed when *tbph* was driven or knocked-down (Diaper et al., 2013). These changes were accompanied by reduced synaptic transmission. There were also decreases in the number of small boutons and impaired BMP signaling at the larval NMJ when human TBPH was driven in neurons (Deshpande et al., 2016). In contrast, *Drosophila dsod1* mutants reveal only subtle changes in larval MNs (Held et al., 2019; Sahin et al., 2017). Because *dsod1^G85R/G85R^* flies show pharate adult lethality with shortened legs and MNs with broken axonal branches, adult MNs appear to be preferentially affected in *dsod1* mutants. To characterize adult MN phenotypes independent of development, we use longer-lived *dsod1^null^* and *dsod1^H71Y^* alleles, which live median life spans of 13.5 and 6.5 days, respectively, and animals develop normally (Sahin et al., 2017). Previously, we showed age-dependent progressive locomotor defects in *dsod1^H71Y/H71Y^* flies (Sahin et al., 2017). Given age-dependent disease progression in ALS, we wondered whether there were neuromuscular changes that correlated with adult locomotor defects in aged *dsod1^H71Y/H71Y^* flies.

### Homozygous *dsod1^H71Y/H71Y^* flies show age-dependent metathoracic leg-dragging phenotype

**Fig. 1.**
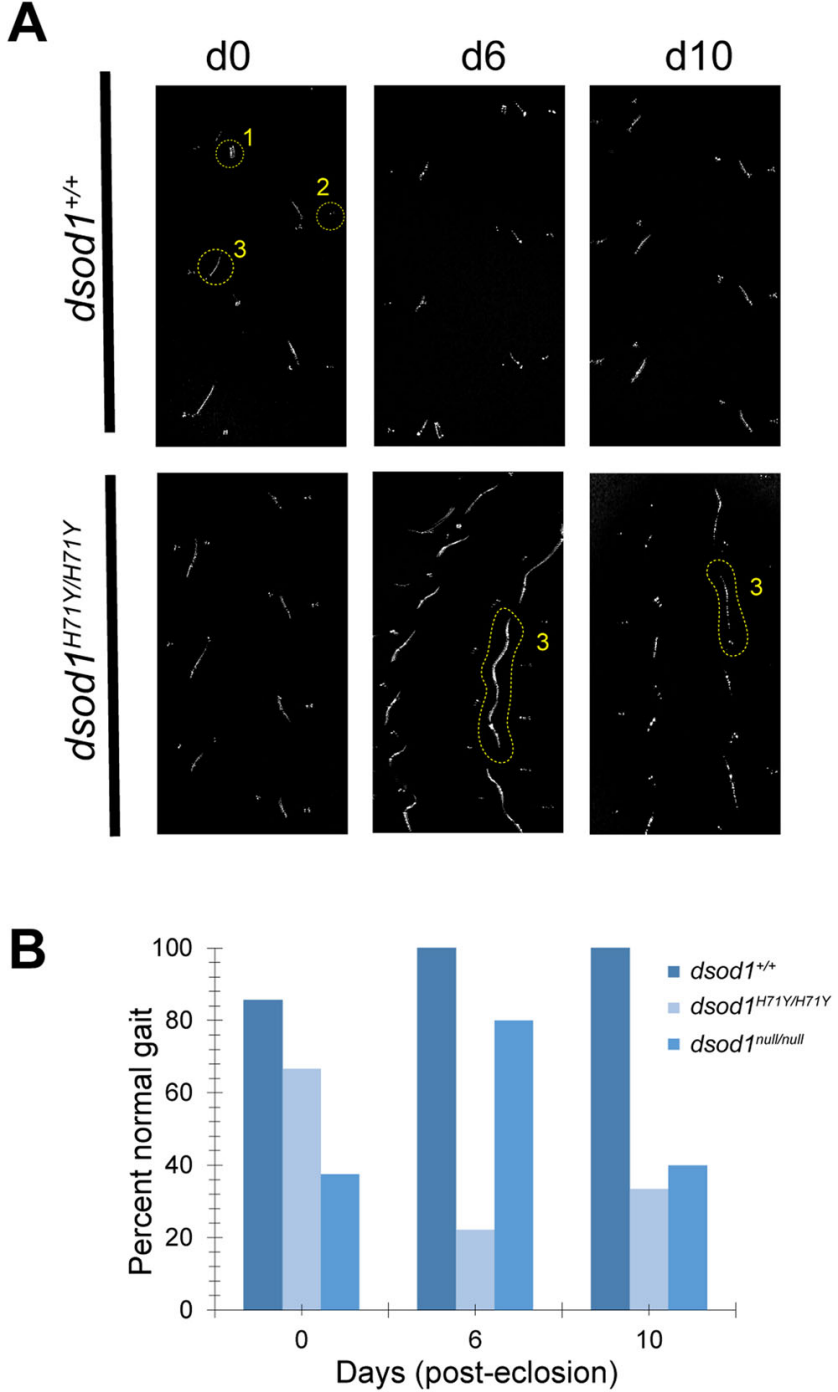
**Age-dependent locomotor defects in *sod* mutants.** (A) Representative images of walking patterns from *dsod1^+/+^*and *dsod1*^H71Y/H71Y^ adults at eclosion (day 0) and in aged flies (days 6 and 10). Footprints resulting from prothoracic (1), mesothoracic (2) and metathoracic legs (3) are labeled in loxP, day 0. Aged *dsod1*^H71Y/H71Y^ mutants display metathoracic leg-dragging phenotypes as shown for days 6 and 10. (B) Percentage of flies displaying normal gait phenotypes is decreased for *dsod1*^H71Y/H71Y^ and *dsod1^null/null^* mutants; *n*=8 (loxP, day 0); 7 (loxP, day 6); 6 (loxP day10); 6 (*dsod1^H71Y/H71Y^*, day 0); 9 (*dsod1^H71Y/H71Y^*, day 6); 6 (*dsod1^H71Y/H71Y^*, day 10); 8 (*dsod1^null/null^*, day 0); 5 (*dsod1^null/null^*, day 6); 5 (*dsod1^null/null^*, day 10).

We assessed adult walking phenotypes using footprinting assays as a measure of locomotor ability in *dsod1* mutants and *dsod1^+^* flies. The *dsod1^+^* genotype used throughout this study contains one loxP site within the single intron of *dsod1^+^* at an identical position found in mutant *dsod1* stocks as a result of ends-out homologous recombination and removal of the mini-*w^+^* selectable marker. The *dsod1^null^* allele was generated by CRISPR and removed the entire ORF as diagramed in Fig. S1. Flies locomote using a repeating tripod or tetrapod gate (Mendes et al., 2013). We analyzed walking patterns for deviations from this repeating pattern and quantified the percentage of flies with either normal or abnormal gait patterns (Fig. 1). Patterns considered abnormal ranged from deviations in pattern spacing to pronounced leg dragging. In newly eclosed adults, >80% of *dsod1^+/+^* flies showed normal walking gaits and this frequency remained high though day 10 aged adults (Fig. 1B). We found lower percentages of flies with normal gaits beginning at eclosion for both mutant *dsod1* alleles. In addition, both *dsod1^H71Y/H71Y^* and *dsod1^null/null^* mutants showed age-related declines in normal locomotion between days 0 to 6 for H71Y (67% to 22%), and days 6 to 10 for null (80% to 40%), respectively. Walking in aged *dsod1^H71Y/H71Y^* was often associated with a prominent metathoracic leg dragging phenotype (as shown in Fig. 1A, *dsod1^H71Y/H71Y^* day 6). Abnormal walking in *dsod1^null/null^* was generally less severe with 0% displaying leg-dragging phenotypes on days 6 and 10 compared to 44% and 17% for *dsod1^H71Y/H71Y^*. These results confirm and extend our initial published observations (Sahin et al., 2017).

### Characterization of axonal arborization for MN-I2

The study of leg MNs is emerging as a model system for the study of slow neurodegenerative processes. In addition, the development of leg MNs through neuroblast lineage analysis and their arborization patterns has been well documented. These studies lay the foundation for further characterization of single neuron populations over time in adults. In ALS, MN populations are differentially affected with fast-fatigable MNs degenerating first followed by fast-fatigue resistant and then slow MNs (Martineau et al., 2018). Thus, there is utility in assessing phenotypes in individual MNs and we focus on the axonal projections from one of two neurons derived from the presumptive ‘I’ neuroblast lineage based on developmental analysis (Baek and Mann, 2009). Both MN-I1 and MN-I2 are glutaminergic neurons that innervate the tibia levitator muscle (TLM) of the femur (Fig. S2). Both MNs were readily identifiable with modest arborization patterns but MN-I2 was less frequently damaged upon dissection in our hands. Based on these practical considerations, we focused on MN-I2 for further analysis.

**Fig. 2.**
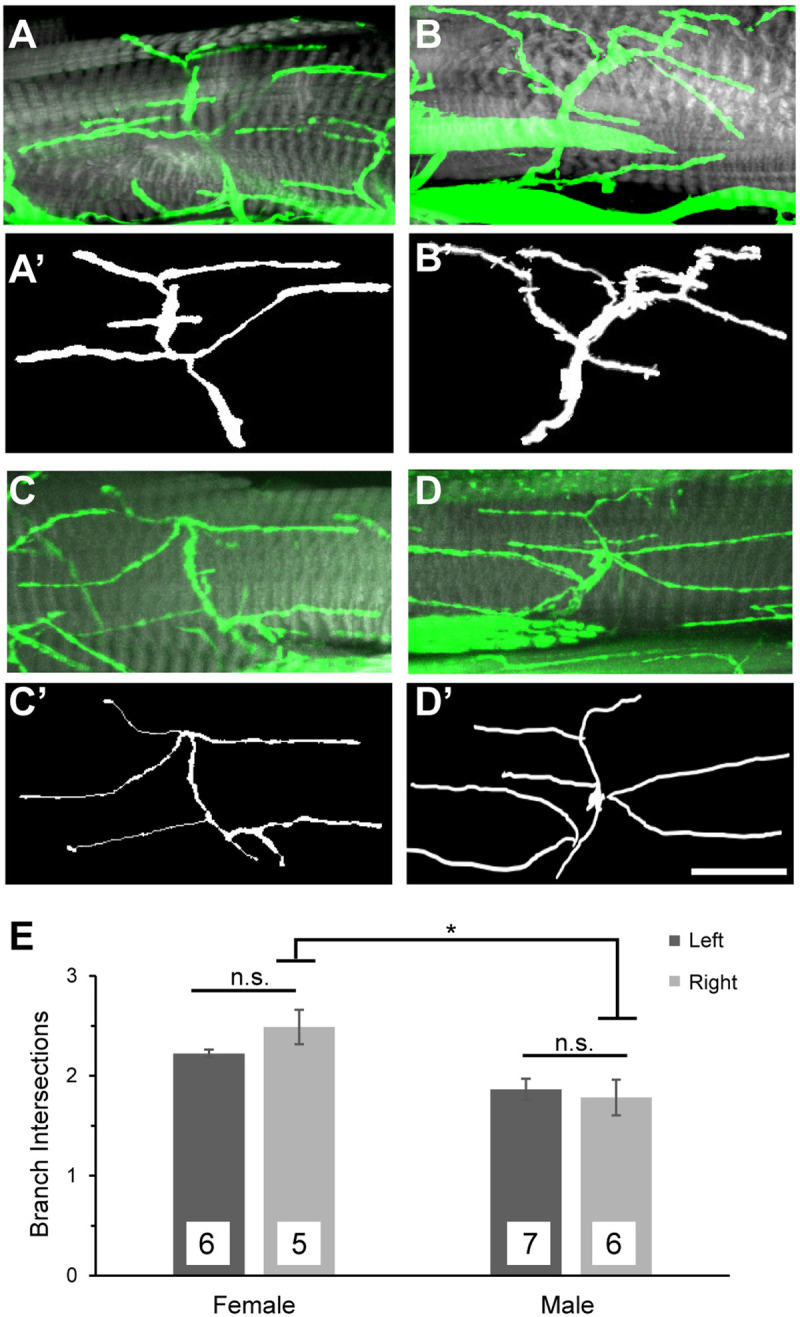
**Characterization of axonal projections of MN-I2 in metathoracic legs.** (A–D) Representative images of MN-I2 from *w^−^; dsod1^+/+^* stocks detected by immunocytochemistry with anti-HRP antibody (green) and phalloidin (gray) in dissected legs of females (A) left and (B) right legs; and males (C) left and (D) right legs. (A′–D′) Corresponding axon tracings used for Sholl analysis performed in ImageJ. (E) Sholl analysis of branch intersections shows a small but statistically significant difference between the right legs of males and females; *P<0.05 (Student's *t*-test). All samples were taken from newly eclosed flies raised at 23°C. *n*=replicate number; scale bar: 20 µm.

To first determine whether MN-I2 makes stereotyped patterns of innervation, we performed Sholl analysis of axon arbors using *dsod1^+^* (loxP) control flies. Because some adult neurons show sexually dimorphic arborization patterns, we compared legs from male and female flies at eclosion (day 0) and representative images and tracings are shown in Fig. 2. While there was no difference in branching intersections between left and right legs, there was a small difference between males and females (*P*<0.05 for the right leg). We further quantified basic parameters of MN-I2 and found reproducible features across individuals including number of branches (7.1±0.7); number of boutons (83.7±9.8) and area of arborization (3898±1010).

### Mutant *sod* flies show age-dependent reduction in arborization

**Fig. 3.**
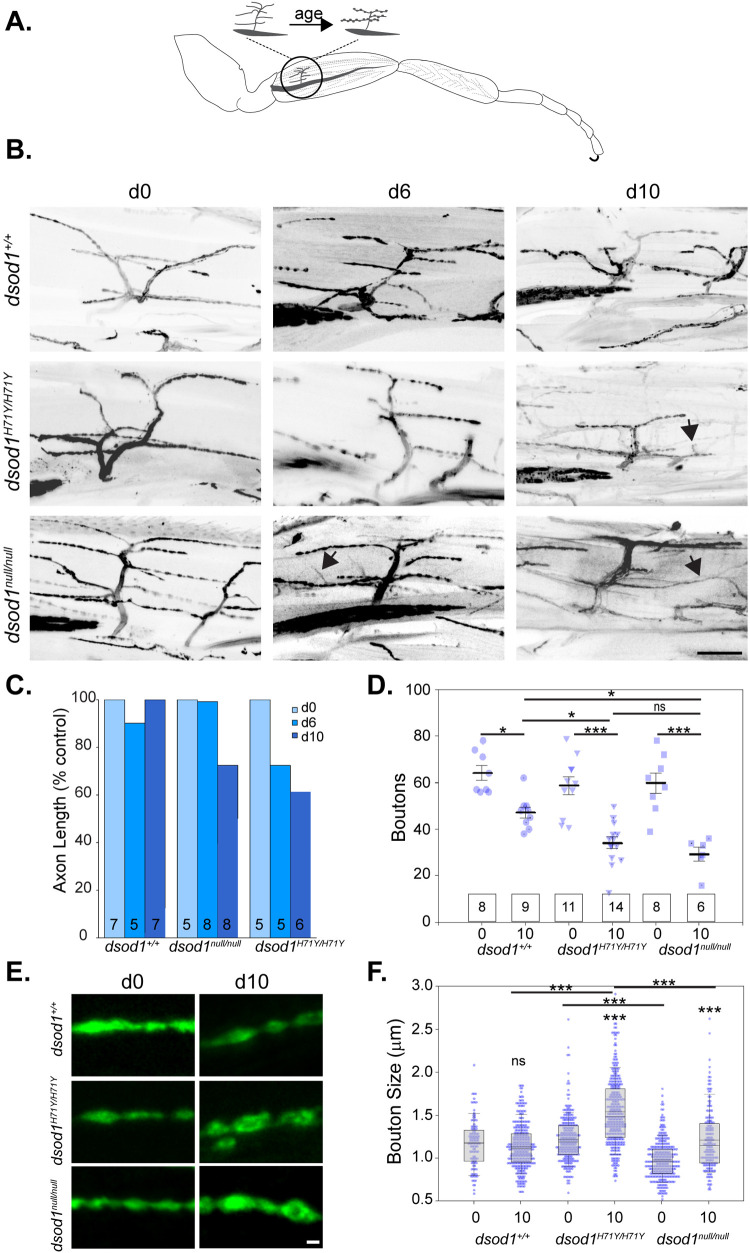
**Age related morphological changes in the MN-I2 axon of *sod* mutants.** (A) Schematic summarizing reduction in branch length and boutons with bouton swelling occurring with age in *dsod1^H71Y/H71Y^* and *dsod1^null/null^* mutants. (B) Representative images of anti-HRP staining for *dsod1^+/+^* and *sod* mutants. Fine arbors which were weakly stained with HRP were apparent in aged *dsod1^H71Y/H71Y^* and *dsod1^null/null^* mutants (arrows, day 6 and 10); scale bar: 20 µm. (C) Age-dependent reduction in total branch length was observed for *dsod1^H71Y/H71Y^* and *dsod1^null/null^*. Quantification of total axon arbor length was expressed as a percentage of respective day 0 controls for each genotype. (D) Number of boutons in MN-I2 decreased with age (0 to 10 days) for each genotype (*P<0.05; ***P<0.0001; two-way ANOVA with post hoc Tukey test); however, there were also significantly fewer boutons in aged *dsod1^H71Y/H71Y^* and *dsod1^null/null^* flies compared to age-matched *dsod1^+/+^*. There were no-significant differences were seen between *dsod1^H71Y/H71Y^* and *dsod1^null/null^* at day 10, and across all genotypes at day 0; sample sizes shown in boxes. (E) Representative boutons imaged from HRP stained preparations show increase in size for aged (day 10) *dsod1^H71Y/H71Y^* and *dsod1^null/null^*. Scale bar: 1 µm. (F) Beeswarm/boxplot showed increased bouton size in day 10 *dsod1^H71Y/H71Y^* and *dsod1^null/null^* relative to day 0, and relative to day 10 *sod^+/+^* (loxP). Bouton size was also increased in day 10 *dsod1^H71Y/H71Y^* compared to *dsod1^null/null^*; ***P<0.0001 (two-way ANOVA with post hoc Tukey test; *n*>250 boutons for each condition.

**Fig. 4.**
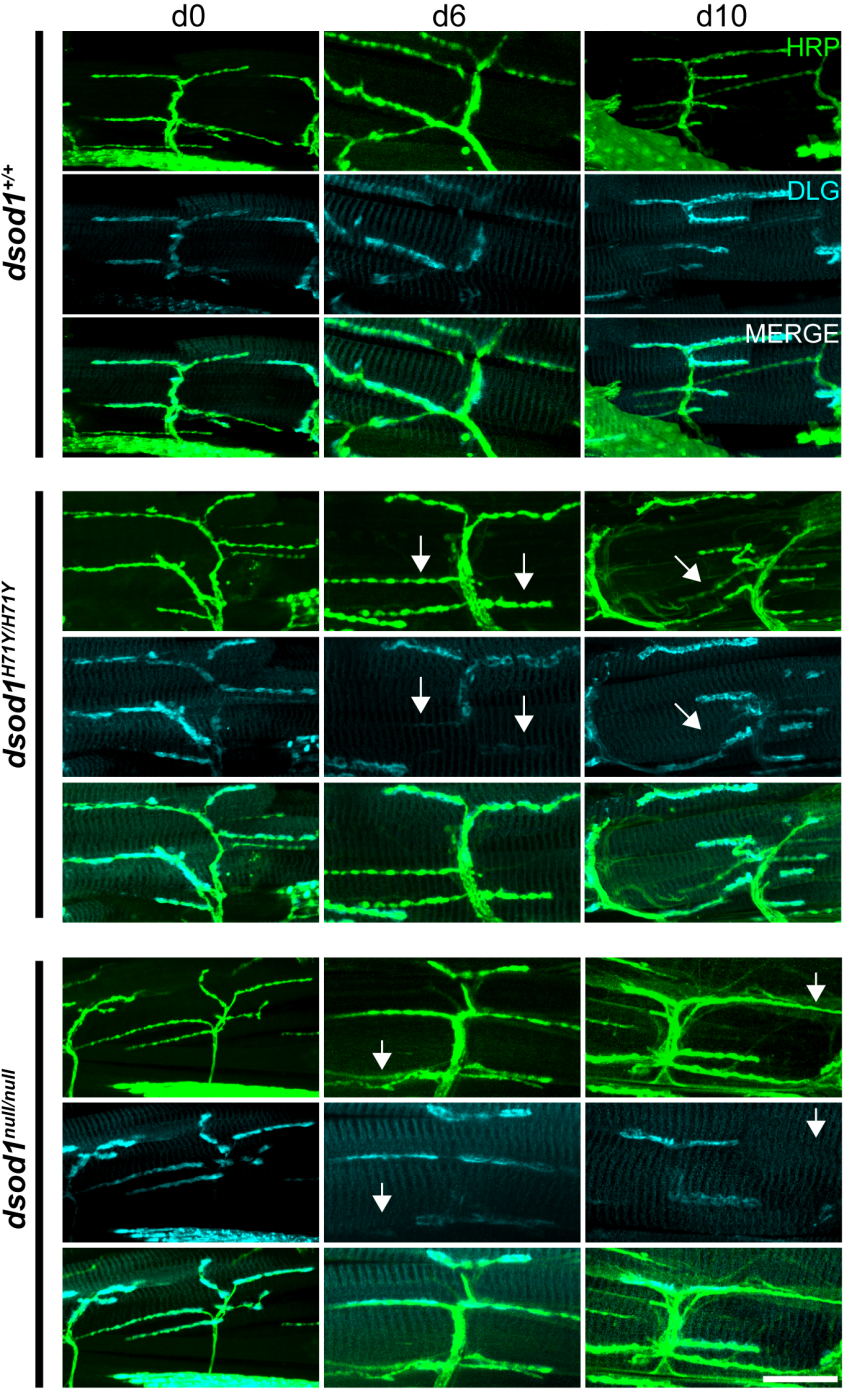
**MN-I2 undergoes age-dependent loss of DLG.** ICC was performed on dissected legs stained with anti-HRP, anti-DLG and phalloidin (blue in merge) and imaged by confocal microscopy. Aged (day 6 and day 10) *dsod1* mutants contain numerous axon branches which lack periplasmic DLG (arrows) compared to aged *dsod1^+/+^* controls. Scale bar: 20 µm.

Reproducible MN-I2 axonal branching allowed us to track changes in innervation patterns over time and we sought to determine whether aspects of dying-back were apparent in the metathoracic legs of ALS-associated *dsod1^H71Y/H71Y^* flies. We also compared MN-I2 characteristics for this allele with *dsod1^null/null^* and *dsod1^+/+^* animals. Newly eclosed *dsod1^H71Y/H71Y^* flies show normal arborization similar to *dsod1^+/+^* (Fig. 3B). Given the relatively short life spans of both mutant *dsod1* alleles, we aged flies over a modest 10-day period. We found *dsod1^H71Y/H71Y^* mutants underwent a retraction in axon projections, which was apparent by day 6 (Fig. 3C). Homozygous *dsod1^null/null^* flies also showed arbor reductions in older flies at 10 days post-eclosion. In addition, the axon arbors of both genotypes appeared disorganized by day 10, lacking a stereotyped pattern with very fine axonal projections (Fig. 3B, arrowheads). In addition, there were broken branches and growth cones or synaptopodia associated with axon sprouting or *de novo* bouton formation as shown in Fig. 4 (Ataman et al., 2008). There was an overall age-dependent reduction in bouton number in aged flies for all genotypes; however, aged *dsod1^H71Y/H71Y^* and *dsod1^null/null^* had fewer boutons than aged *dsod^+/+^* controls (*P*<0.05) (Fig. 3D). Bouton sizes associated with MN-I2 are ∼1.2 µm in *sod^+/+^* flies and this size did not change over a 10-day period. In contrast, we found boutons became significantly enlarged or swollen in *sod^H71Y/H71Y^* and to a lesser extent in *sod^null/null^* mutants (Fig. 3E–G). The similar axonal phenotypes in aged-match *dsod1^H71Y/H71Y^* and *dsod1^null/null^* suggest bouton changes were due to loss of *dsod1* function rather than H71Y associated gain-of-function. Additionally, we found similar branching and bouton sizes in aged *dsod1^H71Y/+^* heterozygotes to age-matched controls that further supports a loss-of-function phenotype (Fig. S3).

### Molecular characteristics of the NMJ in *dsod1* mutants

**Fig. 5.**
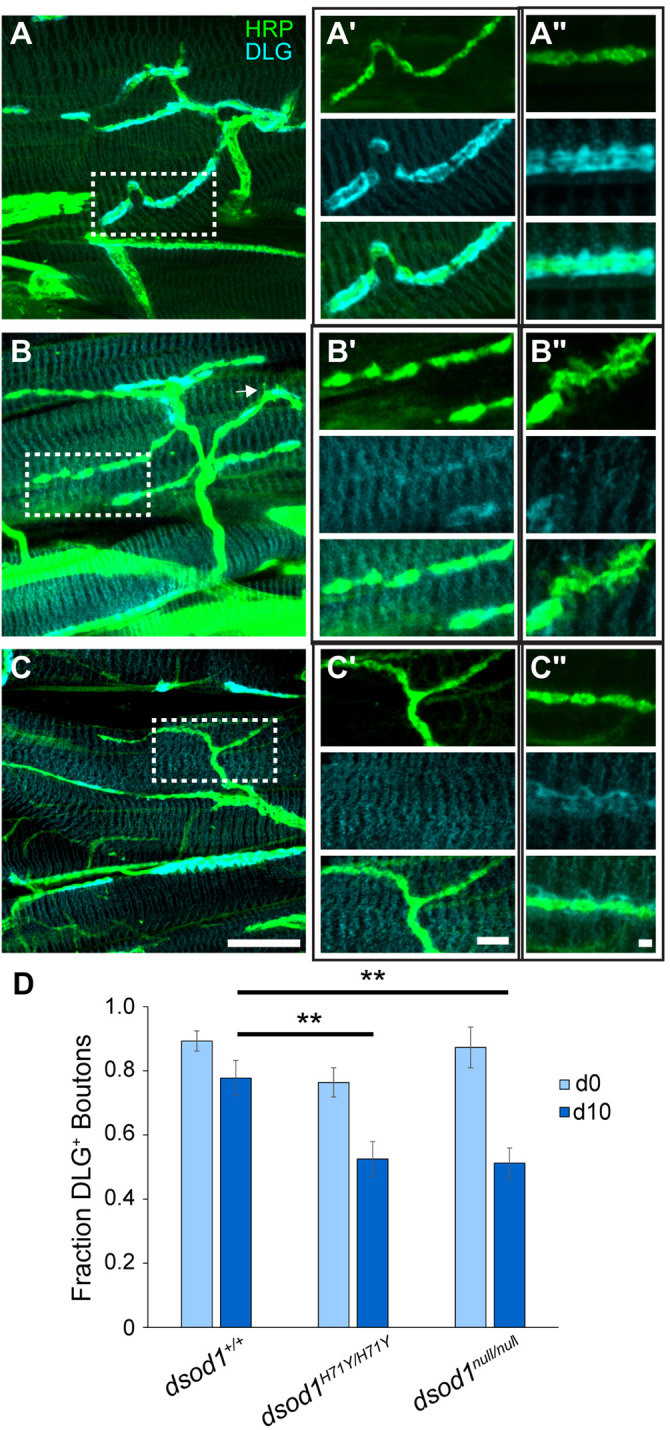
**Loss of DLG is similar between H71Y and null alleles.** Representative HRP and DLG stained preparations in aged (day 10) flies for (A) *dsod1^+/+^*; (B) *dsod1^H71Y/H71Y^* and (C) *dsod1^null/null^*; scale bar: 20 µm. Filopodia-like extensions were observed for *dsod1^H71Y/H71Y^* (arrows) and an additional example shown in B″. These extensions occurred mainly on boutons surrounded by little or no DLG. (A′–C′) Enlarged areas corresponding respective boxed regions in A–C; scale bar: 5 µm. (A′’–C′’) high magnification of representative boutons from separate samples showing reduced or no DLG in sod mutants; scale bar: 1 µm. (D) Individual boutons were scored based on DLG staining relative to background. There were a significantly smaller fraction of boutons with DLG in aged *sod*-mutants relative to d0 controls for each respective genotype (**P<0.01).

In *Drosophila*, functional glutaminergic NMJs contain post-synaptic glutamate receptors clustered by concerted action of several proteins including NETO and DISCS-LARGE (DLG) (Chen and Featherstone, 2005; Kim et al., 2012). To better characterize synapses in *sod* mutants, we performed ICC using anti-HRP and anti-DLG antibodies. Surprisingly, we noticed some of the HRP-staining axonal branches contained boutons that lacked surrounding DLG expression, mostly in aged *sod* mutants (Figs 4 and 5). Ghost boutons that lack DLG in the subsynaptic reticulum have been characterized mainly in larvae and are thought to represent non-functional synapses (Ataman et al., 2008). To quantify the extent of boutons which lacked DLG, we counted DLG positive and negative boutons and found significant (*P*<0.05) decreases in aged *dsod1^H71Y/H71Y^* and *dsod1^null/null^* mutants relative to aged-matched *dsod1^+/+^* as shown in Fig. 5 (53% and 51% for *dsod1^H71Y/H71Y^* and *dsod1^null/null^*, respectively, versus 78% for *dsod1^+/+^*). We also observed statistically significant age-related decreases in *dsod1^null/null^* mutants between newly eclosed and day 10 flies. Lack of associated DLG was found along entire branches and branches that showed bouton-to-bouton variability. Many of the DLG-negative branches also showed weak HRP staining and these boutons appeared to be relatively small (arrows in Figs 4 and 5B and C). Aged *sod^H71Y/H71Y^* mutants were also enriched for boutons with filopodial extensions as shown in Fig. 5B and B″ and were also apparent in fine branches with weak HRP expression (Fig. 4, day 10). We also compared DLG coverage along axonal branches in *dsod1^H71Y/+^* aged flies and found strong DLG expression similar to *dsod1^+/+^* (Fig. S3). Thus age-dependent changes in DLG likely result from dSOD1 loss-of-function.

Flies homozygous for G85R or H48R alleles result in pharate adults that die during adult eclosion or die within 48 h after eclosion, respectively. Given these severe phenotypes, we assessed changes in MN-I2 for G85R and H48R alleles in pharate adults. Flies homozygous for either mutant *dsod1* allele showed highly disorganized MN-I2 branching compared to *dsod1^+/+^* flies. For *dsod1^G85R/G85R^*, there were numerous broken axons (arrow) and also branches that lacked DLG expression (Fig. S4A). Boutons that lacked DLG were quantified and trended lower for G85R, although this difference was not statistically significant (*P*=0.07; Fig. S4B). Finally, we found enlarged boutons relative to controls for both H48R and G85R alleles. Thus, these two most severe *dsod1* alleles also produce early changes in axon morphology.

**Fig. 6.**
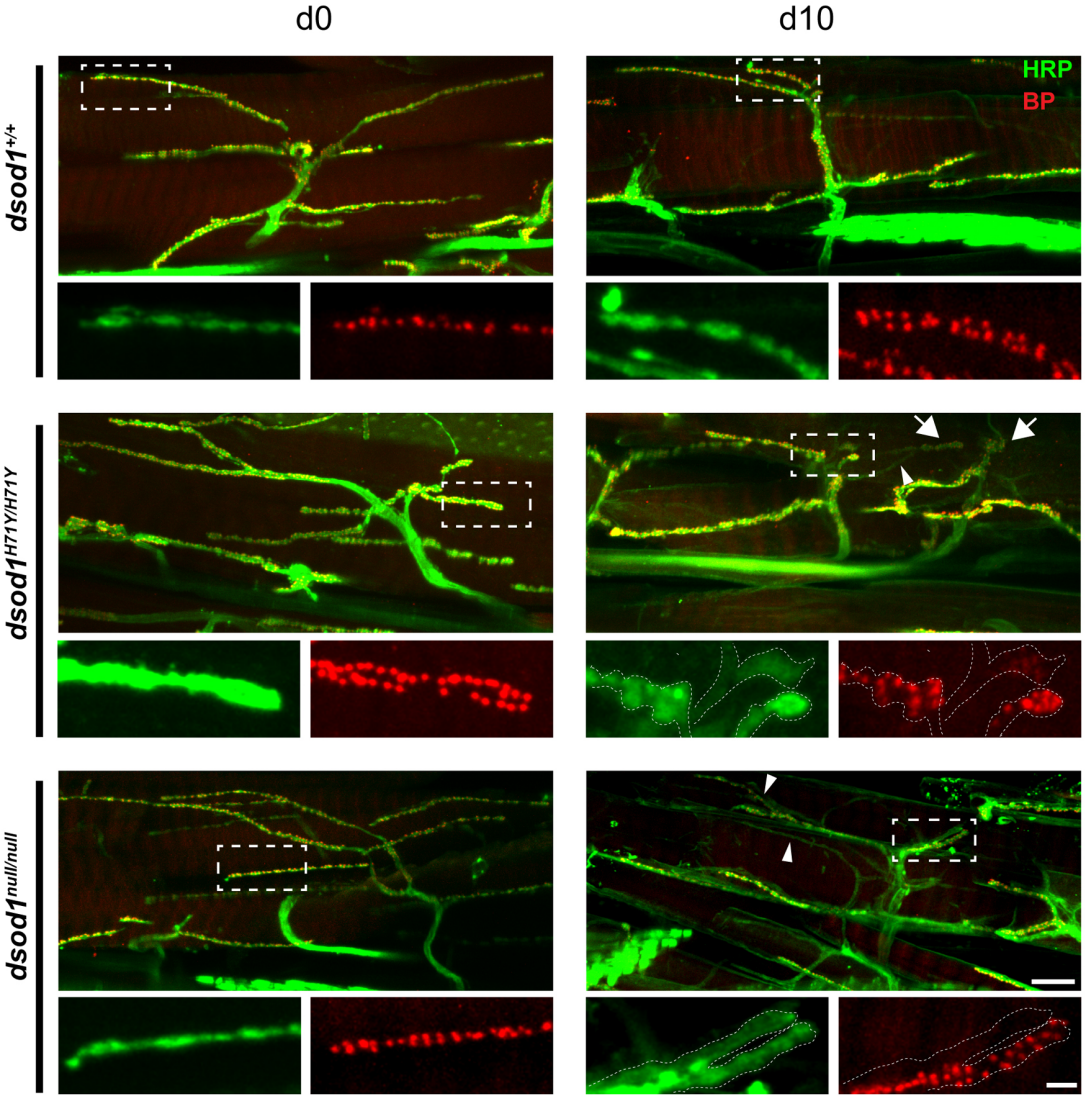
**Alterations in BRP within aged mutant *sod* boutons.** Dissected legs were stained for HRP (green) and the active-zone protein BRP (red) in newly eclosed and 10 day old animals. Swollen varicosities (large arrows) and fine processes (small arrows) enriched in *sod* mutants contained reduced levels and/or diffuse BRP staining; and some axonal processes showed undetectable levels of BRP (as show in inset, for *dsod1*^H71Y/H71Y^ and *dsod1^null/null^* day 10). Scale bars: 20 µm and 2 µm (inset).

We next assessed boutons for active zones across genotypes in aged animals by monitoring bruchpilot (BRP). In *sod^+/+^* animals, BRP was mainly localized to boutons as expected; however, there were also parts of MN-I2 that contained BRP positive puncta but lacked distinct varicosities, including the main MN-I2 axon branch emanating from fascicle. This observation was evident across genotypes and may represent a difference in the location and morphology of NMJs between larval and adult MNs. Across genotypes, we found nearly all morphologically distinct boutons contained BRP-positive active zones; however, very large varicosities present in *sod* mutants showed weak or diffuse BRP staining (for example, see Fig. 6, H71Y inset). Furthermore, fine branches (arrows) that contained few boutons showed little or no BRP staining.

### Mutant *sod* flies show age-dependent increases in poly-ubiquitinated proteins

**Fig. 7.**
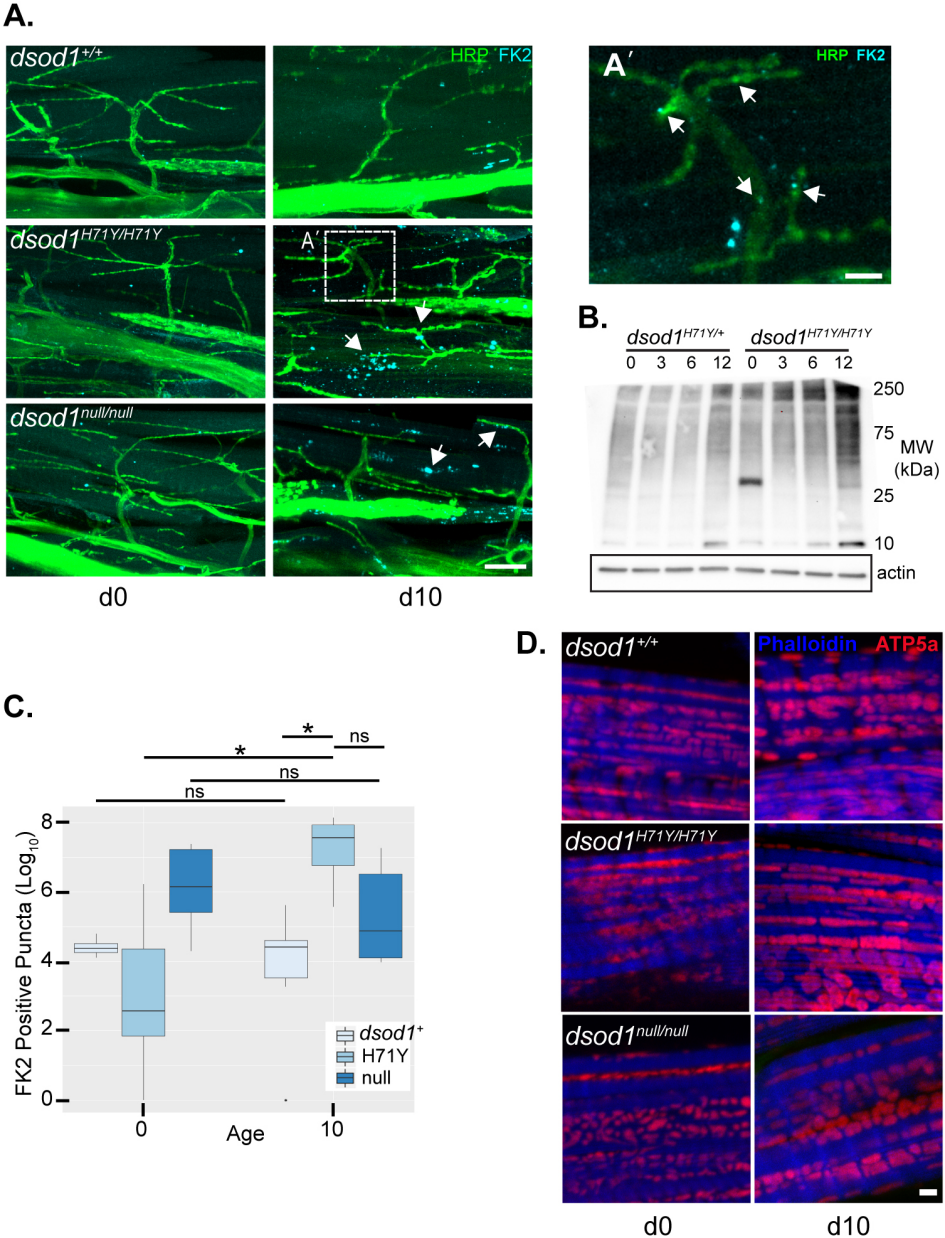
**Polyubiquitin aggregates accumulate in aged *dsod1* mutants.** (A) Representative images from samples stained with HRP (green) and anti-polyubiquitin (FK2, red) show accumulation of FK2 positive puncta (arrows) in aged *dsod1*^H71Y/H71Y^ and to a lesser extent in *dsod1*^null/null^ mutants. Scale bar: 20 µm. (A′) A 5 µm Z-stack showing the MN containing FK2 positive aggregates in distal axons. Scale bar: 5 µm. (B) The number of puncta were quantified using the Analyze Particle plug-in in ImageJ (see the Materials and Methods for more details) and show FK2-positive aggregation is age-dependent in *dsod1*^H71Y/H71Y^ mutants (comparing *dsod1*^H71Y/H71Y^ day 10 versus *dsod1*^H71Y/H71Y^ day 0). The number of polyubiquitinated puncta are also significantly increased in aged *dsod1*^H71Y/H71Y^ compared to *dsod1*^+/+^. *P<0.05; ns, not significant (two-way ANOVA with post hoc Tukey test). *n*=4 (*dsod1*^+/+^, day 0); 10 (*dsod1*^+/+^, day10); 7 (*dsod1*^H71Y/H71Y^, day 0); 9 (*dsod1*^H71Y/H71Y^, day 10); 6 (*dsod1^null/null^*, day 0); 6 (*dsod1^null/null^*, day 10). (C) Representative western blot probed with anti-FK2 for head and thorax tissue from *dsod1*^H71Y/+^ and *dsod1*^H71Y/H71Y^ showing increased polyubiquitinated puncta in aged (day 12) mutant homozygotes. (D) ICC of representative muscle mitochondria stained with ATP5a showing enlarged mitochondria in aged *dsod1*^H71Y/H71Y^ and to a lesser extent, aged *dsod1^null/null^* animals (*n*=5 for each genotype).

An accumulation of polyubiquitinated proteins as a result of decreased proteasome function and autophagy is a characteristic of ALS and other neurodegenerative diseases (reviewed in Blokhuis et al., 2013). We assessed the extent of polyubiquitinated protein aggregation in *Drosophila sod* mutants by performing ICC with an anti-polyubiquitin antibody (FK2) and quantifying puncta. In day 0 adults, there were relatively few FK2 positive puncta present and no statistically significant differences in numbers of puncta across genotypes (Fig. 7A,B). However, FK2 positive aggregates increased in aged *sod^H71Y/H71Y^* versus *sod^+/+^* animals (*P*<0.05). We also found statistically significant increases in FK2-positive puncta between newly eclosed and aged *sod^H71Y/H71Y^* (*P*<0.05). Furthermore, there was an increased in polyubiquitinated aggregates in aged *sod^H71Y/H71Y^* homozygotes compared to *sod^H71Y/+^* heterozygotes (Fig. 7C). We found no differences in polyubiquitinated protein levels between *sod^H71Y/+^* heterozygotes and *sod^+/+^* (loxP) consistent with recessive phenotypes (data not shown). As shown in Fig. 7, puncta are localized to muscle fibers (arrows) but can also be found in distal regions of axons and in boutons (Fig. 7A′). These results indicate that there is an age-dependent accumulation of polyubiquitinated aggregates in *sod^H71Y/H71Y^*. Such aggregates are often associated with changes in mitochondrial morphology and function; and mutant SOD1 are associated directly with mitochondrial membranes (Deng et al., 2006). We assessed age-dependent changes in mitochondrial morphology and observed swollen mitochondria across the TLM muscle in aged versus young *dsod1^H71Y/H71Y^* mutants (Fig. 7D). Furthermore, there were also observed readily apparent changes in aged *dsod1^H71Y/H71Y^* mutants compared to wild-type controls. Mitochondrial swelling in *dsod1^nul/nulll^* animals was also apparent but qualitatively less severe than age-matched *dsod1^H71Y/H71Y^* flies. Interestingly, mitochondria in *sod^G85R/G85R^* homozygotes were similar to controls suggesting morphological changes in mitochondria may be age-related in *dsod1* mutants (Fig. S4D).

## DISCUSSION

This work characterizes changes in the morphology of a single MN that innervates the leg TLM in a *Drosophila* model of ALS. The MN-I2 arborization is less complex than many other adult MNs and possesses modest numbers of branches and boutons, making it easier to quantify changes. Furthermore, as one of several MNs that innervate the TLM, it likely plays an important role in locomotion. We saw subtle differences between MN-I2 arbor complexity in males versus females based on Sholl analysis. Other neuronal classes also show sexually dimorphic morphologies to drive sex specific behaviors (Goto et al., 2011). For leg MNs, sex-specific courtship, mating and egg-laying behaviors necessitate different patterns of innervation (Stockinger et al., 2005). However, it is unclear if or how these differences in branching patterns of MNs affect function. Our characterization showed that MN-I2 forms a stable branching pattern over a 10-day period in *sod^+/+^*adults. While wild-type flies live for ∼90 days, we find ∼35% of *dsod1^H71Y/H71Y^* and ∼60% of *dsod1^null/null^* mutants are alive at 10 days, respectively (Sahin et al., 2017). From eclosion through day 10, *sod* mutants lose locomotor capabilities with a prominent metathoracic leg dragging phenotype. Although many factors contribute to walking behaviors in *Drosophila* including motor, sensory and muscle inputs, there is reasonable correlation between age-dependent deficits in locomotion for *dsod1^H71Y/H71Y^* and reduction in arborization by day 6. Although ∼30% of *dsod1^H71Y/H71Y^* homozygotes showed abnormal walking phenotypes at eclosion, MN-I2 arborization appeared similar to wild-type morphologically. We did not assess neuronal function by electrophysiology, which would provide an important readout, and these studies await future analysis. It should be possible to perform muscle recordings across genotypes and ages to determine the extent to which differences in motor neuron arborizations affect innervation and contractile properties of the muscle. A characterization of neurons innervating leg muscles has been published recently and found that an ordered recruitment of MN activation controls muscle contraction (Azevedo et al., 2020).

ALS is associated with age-dependent denervation. Motor loss begins at presymptomic stages and is associated with concomitant spouting from neighboring axons (Martineau et al., 2018) (Clark et al., 2016) (Hegedus et al., 2007, 2009, 2008). We find bouton loss occurred with age in *dsod1* mutants and occurred along with an overall shortening of total axon length. Furthermore, we observed numerous examples of filopodia, particularly in *dsod1^H71Y/H71Y^* suggesting there may be additional regions of axonal outgrowth. In *Drosophila*, such filopodia are also associated with retraction as seen when the nervous system is remodeled during metamorphosis (Liu et al., 2010). We cannot rule out this possibility given an overall reduction in axon length. During metamorphosis, DLG expression becomes diffuse and boutons become enlarged during NMJ loss. Aged *dsod1* mutants share these characteristics suggesting that NMJs may be similarly dismantled. Interestingly, flies overexpressing wild-type or ALS-associated mutant FUS in MNs show >50% loss of DLG and reduced or diffuse BRP localization within boutons (Machamer et al., 2014). These results may indicate a set of shared molecular events when NMJ destruction is triggered by multiple mechanisms.

While adult MNs undergo age-dependent NMJ loss, larval MNs show only minor defects (Held et al., 2019; Sahin et al., 2017). For *dsod1^G85R/G85R^* mutants, larvae crawl slower but possess similar number of boutons and similar evoked and spontaneous excitatory post-synaptic potentials (eEPSP and sEPSP) compared to wild-type flies. In contrast, MNs innervating the adult abdomen have fewer boutons, fewer sEPSPs, and less periplasmic DLG staining. Because *dsod1^G85R/G85R^* mutants die prior to adult eclosion with morphological leg defects, it is unclear if adult MN development is disrupted or there is rapid NMJ loss after normal outgrowth. Larval MNs may be spared due to maternal dSOD^+^ deposition or because metamorphosis occurs before slow degenerative processes manifest. Alternatively, it is possible that there are molecular and/or physiological differences between adult and larval MN pools making the former differentially susceptible. Other studies have begun to identify stage specific MN properties (Rivlin et al., 2004).

ALS is non-cell autonomous and MN loss involves dysregulation of glia, muscle and MNs. We observed an accumulation of polyubiquitinated aggregates in muscle and distal MN processes. While the majority of ALS studies focus on protein aggregation in brain and spinal cord MNs, muscle aggregates are also detected for TDP-43 and C9ORF72 for both sporadic and familial ALS (Lynch et al., 2019; Mori et al., 2019). Furthermore, muscle-specific expression of mutant SOD1 causes MN degeneration (Dobrowolny et al., 2008; Wong and Martin, 2010). These results argue that muscle plays an important role in disease pathogenesis. Furthermore, maintenance of the NMJ requires both anterograde and retrograde signaling between muscle and MN (James et al., 2019; McCabe et al., 2003; Penney et al., 2016; Budnik and Salinas, 2011). Although not directly assessed in this study, it is possible that pathological events such as dysregulation of retrograde signaling contributes to NMJ loss in *sod* mutant flies. Phosphorylation of DLG through PAR-1 kinase regulates synapse formation and transmission and *dlg* mutant larvae have decreased bouton numbers (Zhang et al., 2007). It is therefore possible that reduction of DLG in *dsod1* mutants causes our observed decreased bouton numbers in MN-I2. There are also defects in MN retrograde signaling at presymptomic stages in ALS models (Gibbs et al., 2018).

There is a strong association between SOD1 protein aggregation potential and ALS progression for the spectrum of *sod1* gene mutations and a good correlation exists between aggregation potential and survival times across a broad spectrum of *sod1* mutations (Lang et al., 2015). However, most *sod1* mutations also decrease enzymatic activity and enzyme half-life with varying degrees (Borchelt et al., 1994; Saccon et al., 2013). Interesting, mutant SOD1^A4V^ retains some enzymatic activity yet causes an aggressive form of ALS. In CRISPR-edited human cells and humanized yeast strains, heterozygous SOD1^A4V/+^ cells showed increased lipid peroxidation indicative of oxidative damage and substantial aggregate formation despite retaining SOD1 activity (Brasil et al., 2019). These data along with numerous other reports show aggregation and oxidative stress are linked. Thus, there is apparent oxidative damage even when SOD1 retains enzymatic activity. Mutant SOD1-H71Y protein disrupts Zn^2+^ binding critical for enzymatic activity rendering the mutant protein inactive (Cruz-Garcia et al., 2017). We have confirmed lack of enzymatic activity previously in *sod^H71Y/H71Y^ Drosophila* (Sahin et al., 2017). Thus, increased oxidative damage associated with loss of SOD1 function likely contributes directly to NMJ pathology for this and other disease-linked *sod* alleles where enzymatic activity is reduced. Similar studies describe a distal axonopathy in SOD1-knockout mice, which includes terminal axon swelling aberrant sprouting and phosphorylated neurofilament accumulation (Fischer et al., 2012). The fact that we observe similar NMJ phenotypes between *sod^H71Y/H71Y^* and *sod^null/null^* flies indicates that oxidative damage resulting from loss-of-function likely plays a significant role in NMJ pathology. However, we did find a few notable phenotypic differences between H71Y and null alleles. In *sod^H71Y/H71Y^* flies, frequency of leg-dragging was greater, arborization decreases occurred earlier, and bouton sizes were larger relative to age-matched *sod^null/null^*. Thus, our data indicate a combination of gain- and loss-of-function phenotypes in *sod^H71Y^/sod^H71Y^* flies. Other groups have suggested that ALS caused by *sod1* mutations is due to both gain and loss-of-function and our data generally support this model (Baskoylu et al., 2018; Saccon et al., 2013; Sau et al., 2007).

Here we use a novel leg dissection preparation to characterize the axon arbor from a single MN in *Drosophila*. The MN-I2 motor neuron is easily identifiable and tractable to morphometric and molecular analysis making further studies of aging and slow degenerative processes possible. In *dsod1* mutant adults, we show that MN-I2 undergoes an age-dependent reduction in axon length accompanied by a loss of boutons. Boutons remaining in MN-I2 are enlarged and periplasmic DLG is reduced or absent. Finally, we find an accumulation of polyubiquitinated aggregates in aged flies. Although overt MN loss does not occur in mutant *sod* flies, these observations are consistent with denervation as seen in other ALS model systems and confirms the robustness of our model in recapitulating important cellular aspects of human ALS.

## MATERIALS AND METHODS

### Fly stocks and husbandry

*Drosophila sod* alleles were described previously (Sahin et al., 2017). Fly stocks were reared on standard sucrose, cornmeal, agar media at 23°C with a 12:12 light:dark cycle. For aging experiments, newly eclosed flies were collected daily and changed to new media every 1–3 days.

### Footprinting assays

Adult walking assays were performed as previously described (Diaper et al., 2013). Images were captured with a compound microscope (100x) and brightness and contrast were adjusted as needed.

### Immunohistochemistry

Metathoracic legs were cut at the coxal segment and fixed in 4% formaldehyde for 45 min prior to dissection. After washing 3× in PBS, a cuticle patch along the femur was removed exposing underlying tissue and samples were post-fixed for 15 min in 4% formaldehyde. Antibody staining protocols followed standard methods and samples were mounted in slow-fade diamond mount (Molecular Probes) (Patel, 1994). Antibodies and stains used were anti-horse-radish peroxidase (#115-035-166, Jackson ImmunoResearch, 1:550), anti-dlg (4F3, Developmental Studies Hybridoma Bank, 1:200), phalloidin-633 (Molecular Probes, 1:1000), mouse anti-FK2 monoclonal (#BML-PW0150-0025, Enzo Life Sciences, 1:200); mouse anti-Bruchpilot monoclonal (nc82, Developmental Studies Hybridoma Bank, 1:20), mouse anti-ATP5a (#ab14748, Abcam, 1:200). Imaging was performed by confocal microscopy (Olympus FV1000) capturing z-stacks for all samples. Image processing occurred in ImageJ (FIJI) and consisted of cropping images for areas of interest and altering brightness/contrast as needed for publication. Where necessary image size was increased using linear transformation. Image stitching was performed in ImageJ as needed (Preibisch et al., 2009).

### Quantification of morphology

Sholl analysis was performed on MN-I2 to assess differences in motor neuron structure as described previously (Ferreira et al., 2014; Sholl, 1953). Briefly, the center was specified using the straight-line method starting at the branch point where MN-I2 exits the main axonal bundle and the ends at the most distal part of the motor neuron. The average number of intersections was recorded for left and right metathoracic legs for male and female recently eclosed flies. Student's *t*-test was performed between left/right and male/female samples.

### Quantification of morphological features

Measurement of axon lengths were performed with the Simple Neurite Tracer plugin for ImageJ (Longair et al., 2011). Bouton counts and sizes were also determined within ImageJ using the count and measure functions, respectively. Data were evaluated by Levene's test for homogeneity of variance and Shapiro's test for assessing normality. Bouton sizes were transformed using natural-log transformation to normalize the distribution of bouton sizes. For bouton counts, sizes, and axon lengths a two-way ANOVA with a post hoc Tukey test was performed with respect to age and genotype to account for multiple comparisons.

### Western blotting

Western blotting was performed as previously described (Sahin et al., 2017). Briefly, metathoracic legs were isolated and homogenized at 4°C in 40% RIPA, 60% lammeli+5% β-mercaptoethanol. The samples were spun for 10 min at 16,000×***g*** and the supernatant was transferred to a new microcentrifuge tube. The supernatant was then boiled for 10 min, cooled on ice and then loaded onto 4–20% SDS-PAGE gels. Following electrophoresis, protein was transferred to blotting membrane, and blocked for 2 h at room temperature in superblock (Thermo Fisher Scientific) or 1% BSA in tris-buffered saline +0.01% Tween (TBST). Following primary antibody incubation and wash, blots were incubated with anti-peroxidase secondary antibodies (Jackson ImmunoResearch,1:10,000) for 2 h and washed three times in TBST. Blots were developed with SuperSignal West Pico Plus Chemiluminescence (Thermo Fisher Scientific) and imaged using a Chemidoc digital imaging system (Bio-Rad).

### Puncta analysis

Polyubiquitin-positive puncta detected by ICC were quantified across genotypes and ages using the Analyze Particle plugin within ImageJ on 1400 µm areas encompassing MN-I2 using images captured by confocal microscopy at the same magnification (60× objective with 1.4× zoom).

## Supplementary Material

Supplementary information
