# Supplementary material for: Age-dependent degeneration of an identified adult leg motor neuron in a Drosophila SOD1 model of ALS
Source: Biol Open. 2020 Oct 21;9(10):bio049692. doi: 10.1242/bio.049692 (PMC7595701; doi:10.1242/bio.049692)
Supplement: Supplementary information [file biolopen-9-049692-s1.pdf]

Figure S1

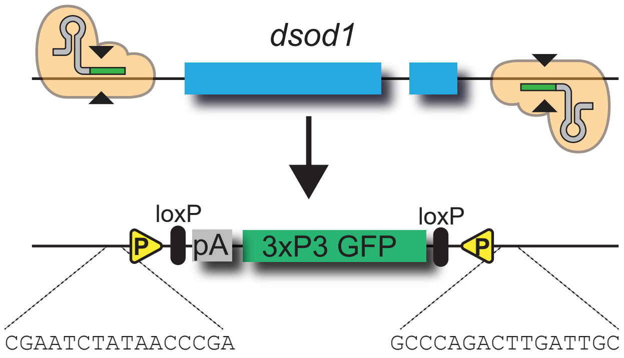

Figure S1. Diagram of *dsod1*^{null} allele. Crispr-mediated deletion of *dsod1* was performed using gRNAs described previously (Sahin et al., 2017). The *dsod1* ORF was replaced with a targeting cassette containing GFP and attB sites. Flanking genomic sequences are shown defining the insertion sites Chr3L:11,112,198 and Chr3L:11,114,063 for left and right arms.

Biology Open • Supplementary information

Figure S2

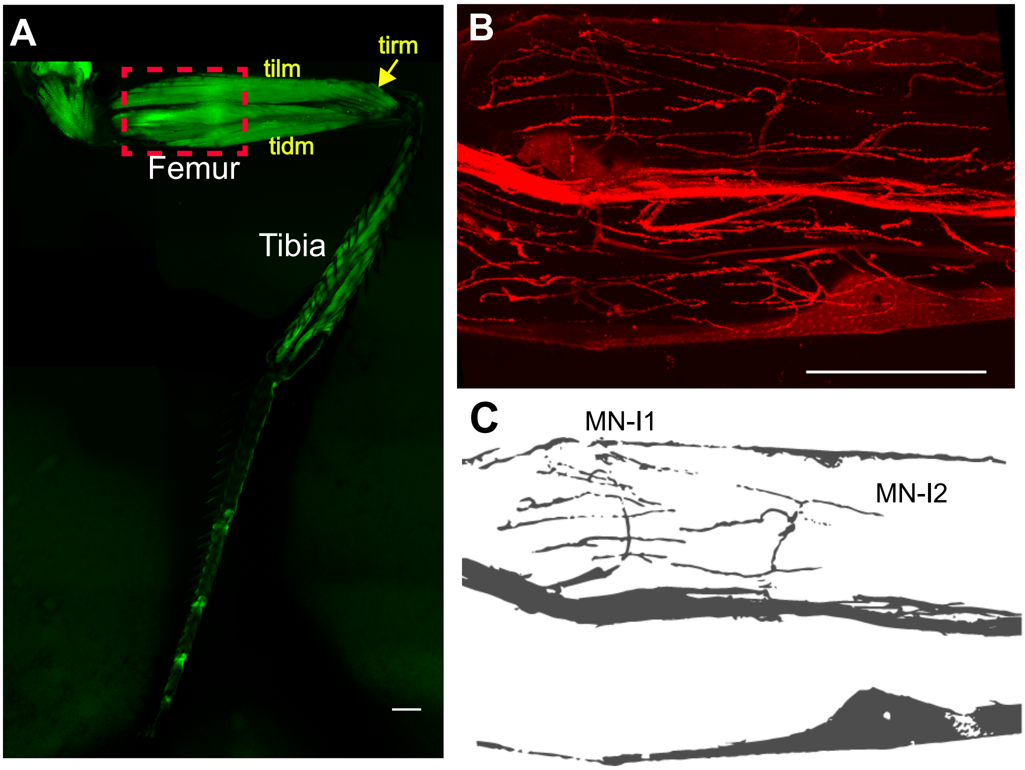

Figure S2. Identification of MN-I2. (A) Confocal image of musculature in a metathoracic leg of MHC-gal4> UAS- GFP expressing flies showing the femur and tibia segments along with the tibia levitator (TILM), tibia depressor (TIDM), and tibia retractor (TIRM) muscles. Boxed region shows location of MN-I1 and MN-I2. (B) Glutaminergic expressing neurons in D42-gal4>UAS-RFP flies. Legs were dissected, mounted in Diamond mount and imaged directly. (C) Tracings showing MN-I1 and MN-I2 created using the trace function in Adobe Illustrator and removing other neurons and branches.

Figure S3

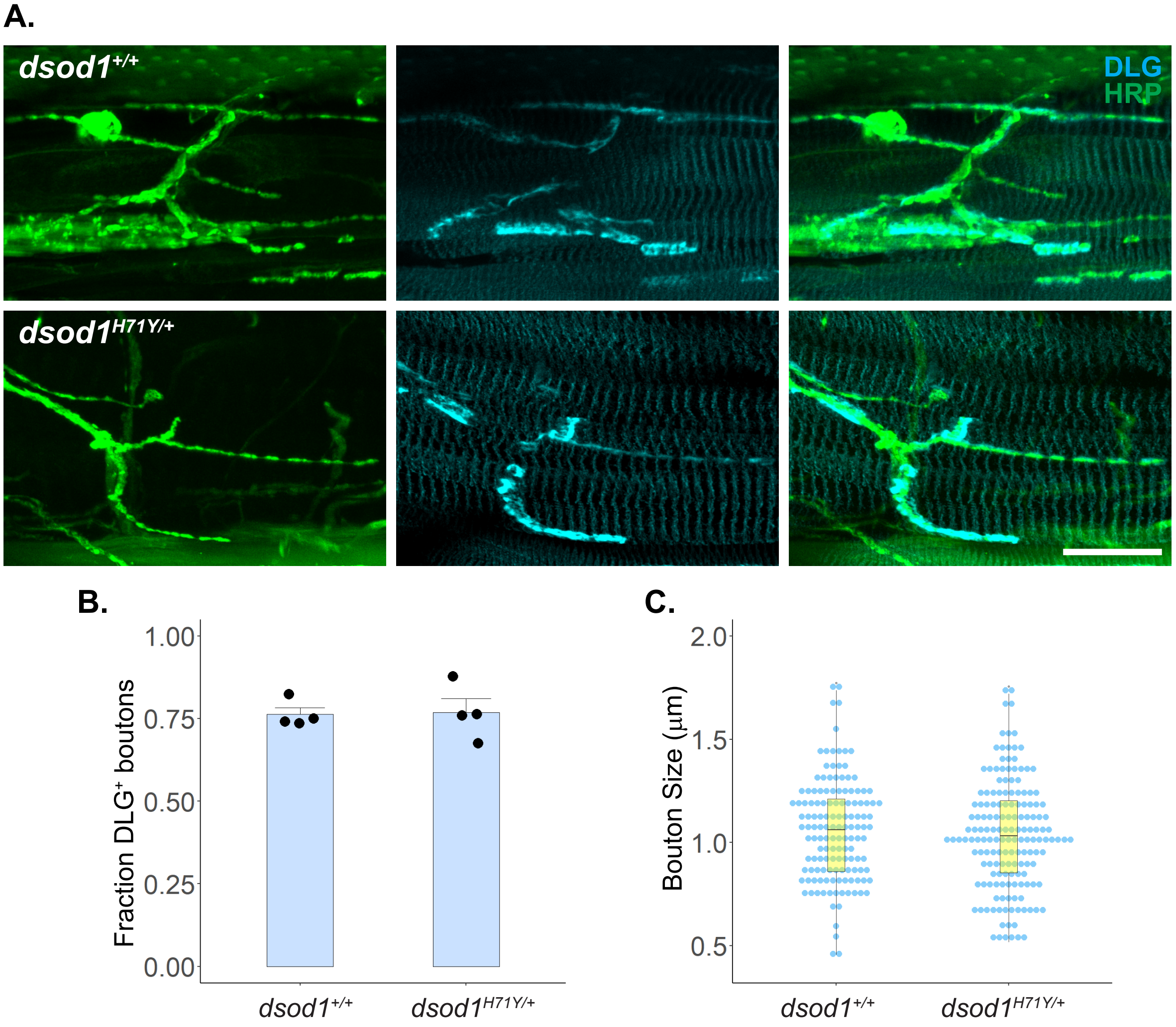

Figure S3. Heterozygous *dsod1$^{H71Y/+}$* and wild type MNs are morphologically similar. (A) Compressed confocal stacks showing representative motor neuron arborization and DLG expression for MN-I2 in aged flies (n=4). (B) Quantification of DLG coverage based on images in A. (C) Bouton sizes in aged animals. Scale bar is 20 µm.

Biology Open • Supplementary information

Biology Open (2020): doi:10.1242/bio.049692: Supplementary information

Figure S4

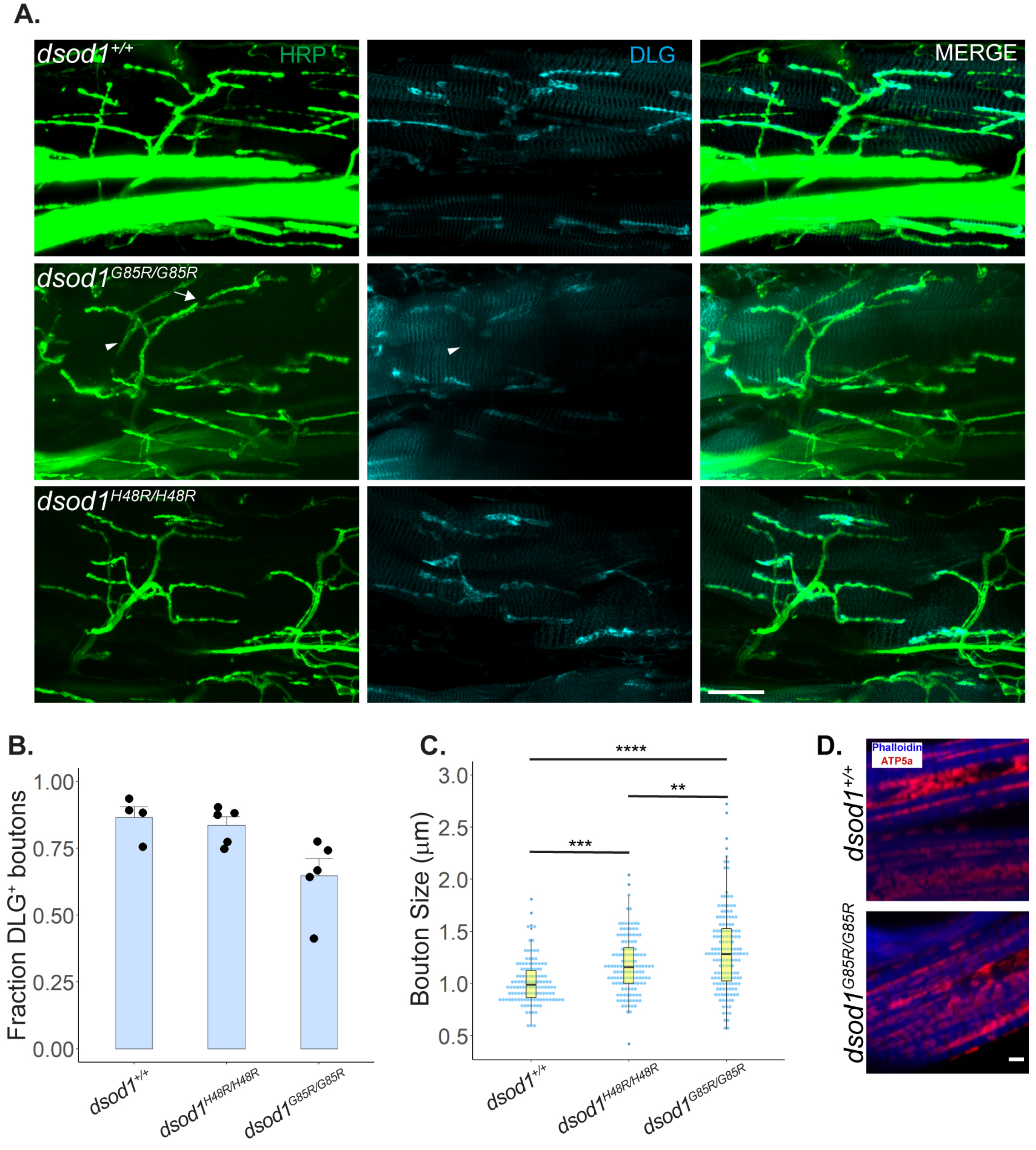

Figure S4. G85R and H48R alleles show early MN-I2 defects in pharate adults. (A) Representative MN-I2 arborization and DLG expression for *dsod1* alleles in pharate adults show highly disorganized arbors with broken branches in *dsod1*$^{G85R/G85R}$ animals (arrow) and branches lacking DLG expression (arrowhead). Scale bar =20 µm. (B and C) Quantification of DLG coverage and bouton sizes. **p<0.001, ***p<0.0005; ****p<0.0001. D) Dissected samples stained with the mitochondria specific marker ATP5a and representative TILM muscle mitochondria are shown (n=5 for each genotype). Scale bar =2 µm.

Biology Open • Supplementary information
